# Substrate adaptors are flexible tethering modules that enhance substrate methylation by the arginine methyltransferase PRMT5

**DOI:** 10.1016/j.jbc.2025.108165

**Published:** 2025-01-08

**Authors:** Cyrus Y. Jin, Moritz Hunkeler, Kathleen M. Mulvaney, William R. Sellers, Eric S. Fischer

**Affiliations:** 1 Department of Cancer Biology, Dana-Farber Cancer Institute, Boston, Massachusetts, USA; 2 Department of Biological Chemistry and Molecular Pharmacology, Harvard Medical School, Boston, Massachusetts, USA; 3 Fralin Biomedical Research Institute, Virginia Tech FBRI Cancer Research Center, Washington, District of Columbia, USA; 4 Cancer Program, Broad Institute of MIT and Harvard, Cambridge, Massachusetts, USA; 5 Department of Medical Oncology, Dana-Farber Cancer Institute, Boston, Massachusetts, USA

**Keywords:** cryo-EM, protein arginine methyltransferase 5 (PRMT5), protein methylation, arginine methylation, spliceosome

## Abstract

Protein arginine methyltransferase (PRMT) 5 is an essential arginine methyltransferase responsible for the majority of cellular symmetric dimethyl-arginine marks. PRMT5 uses substrate adaptors such as pICln, RIOK1, and COPR5 to recruit and methylate a wide range of substrates. Although the substrate adaptors play important roles in substrate recognition, how they direct PRMT5 activity towards specific substrates remains incompletely understood. Using biochemistry and cryogenic electron microscopy, we show that these adaptors compete for the same binding site on PRMT5. We find that substrate adaptor and substrate complexes are bound to PRMT5 through two peptide motifs, enabling these adaptors to act as flexible tethering modules to enhance substrate methylation. Taken together, our results shed structural and mechanistic light on the PRMT5 substrate adaptor function and the biochemical nature of PRMT5 interactors.

Arginine methylation is a posttranslational modification that results in the addition of one or two methyl groups to the guanidino moiety of arginine from the cofactor S-Adenosyl-L-methionine (SAM) (1, 2, 3). Among the three types of protein arginine methyltransferases (PRMTs), type II PRMTs are responsible for installing symmetric dimethyl-arginine (SDMA) marks (4, 5). Type II PRMTs include PRMT5 and PRMT9, where PRMT5 is the main writer of cellular SDMA (6). PRMT5 deposits SDMA marks on a variety of substrates such as the histone tails (H4R3, H3R8), ribosomal protein S10 (RPS10), and spliceosomal proteins SmD1 and SmD3/B, thereby regulating numerous processes involved in cellular gene expression (7, 8, 9, 10). Multiple classes of PRMT5 inhibitors, such as substrate and SAM competitive inhibitors, have been developed (11, 12, 13, 14). Importantly, PRMT5 was identified as a synthetic lethal interaction in the context of methylthioadenosine phosphorylase (MTAP)-depleted cancers, where MTAP deletion results in buildup of methylthioadenosine (MTA), partially inhibiting PRMT5 activity in a selective manner and thus rendering cells sensitive to further PRMT5 inhibition. These findings led to the development of MTA cooperative inhibitors as a new class of PRMT5 inhibitors now in active clinical development (15, 16). pICln and RIOK1, two substrate adaptors for PRMT5 enabling methylation of SmD1/D3/B and RPS10, respectively, also scored significantly as synthetic lethal interactions in MTAP-depleted cancers, underscoring the importance to understand substrate adaptor function in the context of PRMT5 activity and potential resistance mechanisms in PRMT5-dependent cancers (17, 18, 19, 20). Overall, the wide range of cellular processes regulated by PRMT5 and their clinical relevance emphasizes the importance to understand PRMT5 function and regulation at a molecular level.

Much insight into these questions emerged from biochemical, biophysical, and structural studies of PRMT5. Previous structural analysis revealed that PRMT5 forms a hetero-octamer with its obligate binding partner MEP50/WDR77. The N-terminal TIM barrel of PRMT5 binds to WDR77 and facilitates oligomerization with other PRMT5 protomers (21, 22). The TIM barrel also engages substrate adaptors of PRMT5 through their PRMT5 binding motif (PBM) peptide (23, 24). Moreover, the C-terminal catalytic domain of PRMT5 preferentially recognizes and methylates glycine- and arginine-rich motifs (25, 26).

For at least 40% of PRMT5 substrates, adaptor proteins are integral for promoting their methylation (23). pICln forms a complex with SmD1/D2/E/F/G—the 6S complex—to enable methylation on the C-terminal GRG tail of SmD1 (27, 28, 29, 30, 31). This methylation is essential for spliceosome formation and subsequent splicing of a class of retained introns known as detained introns (24, 32). pICln binding to Sm proteins acts as a kinetic trap to prevent illicit formation of Sm complexes on cognate small nuclear RNA (33, 34). The SMN complex is subsequently able to recognize the methyl marks and act as a chaperone to facilitate snRNP formation and functional mRNA splicing (35, 36, 37, 38, 39, 40).

While the cellular importance of PRMT5 substrate adaptors for substrate methylation has been established, mechanistic and structural details are not as clear. Here, we demonstrate that different PBM peptide containing adaptors compete for binding to the PRMT5 methylosome. Through cryogenic electron microscopy (cryo-EM), we find that adaptor and substrate proteins interact with PRMT5 through the PBM peptide and GRG tail, respectively, in a flexibly tethered manner. We observed that binding of the adaptor to PRMT5 allows for nuanced modulation of substrate methylation. Taken together, our studies provide a biochemical and structural basis for the nature of PRMT5 interactors and how adaptors are utilized to enable substrate methylation.

## Results

### Specific peptide motifs mediate adaptor and substrate recruitment

**Figure 1:**
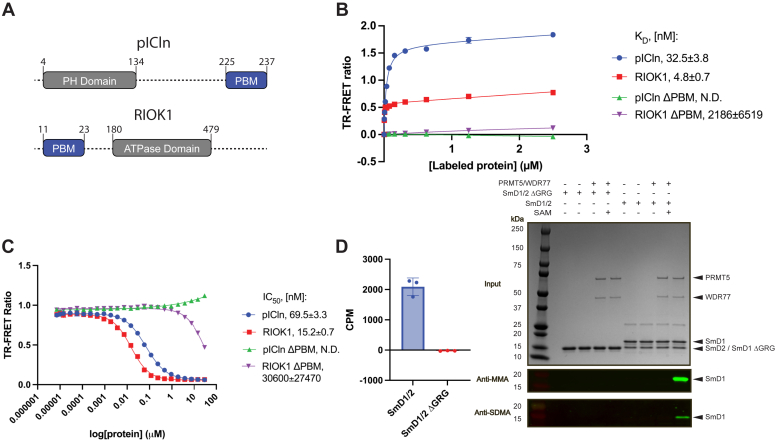
**PRMT5 adaptors compete for binding through their PBM peptide.***A*, domain schematic of pICln and RIOK1. *Dashed lines* indicate predicted flexible regions. *B*, adaptors labeled by BODIPY-maleimide were titrated into PRMT5/biotinylated WDR77 complex incubated with terbium-coupled streptavidin. Equilibrium binding constants for pICln, RIOK1, pICln ΔPBM, and RIOK1 ΔPBM were 32.5 ± 3.8, 4.8 ± 0.7, undetectable, and 2186 ± 6519 nM, respectively. Data is represented as mean ± SD from three technical replicates. *C*, TR-FRET displacement assay displacing BODIPY-labeled pICln from PRMT5/WDR77. IC_50_ values for pICln, RIOK1, pICln ΔPBM, and RIOK1 ΔPBM were 69.5 ± 3.3, 15.2 ± 0.7, undetectable, and 30,600 ± 27,470 nM, respectively. Data is represented as mean ± SD from three technical replicates. *D*, *Left:* methylation SPA of biotinylated full-length SmD1/2 and SmD1 ΔGRG/SmD2. Data is represented as mean ± SD from three technical replicates. *Right:* Western blot against mono methyl and symmetric dimethyl-arginine of full-length SmD1/2 and SmD1 ΔGRG/SmD2 methylated by PRMT5/WDR77. PBM, PRMT5 binding motif; PRMT, protein arginine methyltransferase; SPA, scintillation proximity assay; TR-FRET, time-resolved FRET.

To characterize the binding of pICln and RIOK1 adaptors—which feature PBM peptides on their C- and N-terminal regions, respectively (Fig. 1*A*)—with the PRMT5 methylosome, we established a time-resolved FRET (TR-FRET)-based equilibrium binding assay. Full-length recombinant RIOK1 displayed the strongest binding, with a dissociation constant (*K*_d_) of 4.8 ± 0.7 nM, followed by pICln with a *K*_d_ of 32.5 ± 3.8 nM, consistent with the rank order established with peptides containing the isolated binding motif (23). Truncation of the PBM peptide from RIOK1 and pICIn rendered these adaptors incapable of binding to the PRMT5–WDR77 complex (Fig. 1*B*). To examine the binding further, we established a TR-FRET displacement assay using BODIPY-pICln as a tracer, which allowed us to titrate in unlabeled pICln, RIOK1, pICln ΔPBM, and RIOK1 ΔPBM. We observed IC_50_ values of 69.5 ± 3.3 and 15.2 ± 0.7 nM for pICln and RIOK1, respectively, while the ΔPBM constructs did not exhibit meaningful displacement of the tracer (Fig. 1*C*). These biochemical experiments confirm the importance of the PBM peptide in mediating substrate adaptor recruitment to PRMT5 and suggest that adaptors compete for the same binding site on the PRMT5 methylosome without the requirement for an exchange factor.

We also examined methylation of the substrate protein SmD1/2 in absence of adaptor to test whether the presence of a C-terminal stretch of GRG motifs on SmD1, the canonical motif implicated in PRMT5 mediated methylation, is sufficient for activity (41). We developed a scintillation proximity assay (SPA) to measure methylation of SmD1 by PRMT5 using biotinylated SmD1/2 complex. We observed a lack of detectable methylation on SmD1 when the C-terminal GRG motif was truncated. This was corroborated by a lack of methylation in a Western blot detecting mono methyl and symmetric dimethyl-arginine marks (Fig. 1*D*). The lack of activity with SmD1 ΔGRG demonstrates the importance of the GRG peptide stretch as the site of methylation between PRMT5 and substrate and suggests that at least *in vitro* basal activity can be achieved in absence of adaptors.

Taken together, these studies highlight the importance of specific peptide motifs in adaptor and substrate proteins for establishing interactions and promoting activity of the PRMT5 methylosome.

### Structural characterization of a PRMT5–adaptor–substrate complex

**Figure 2:**
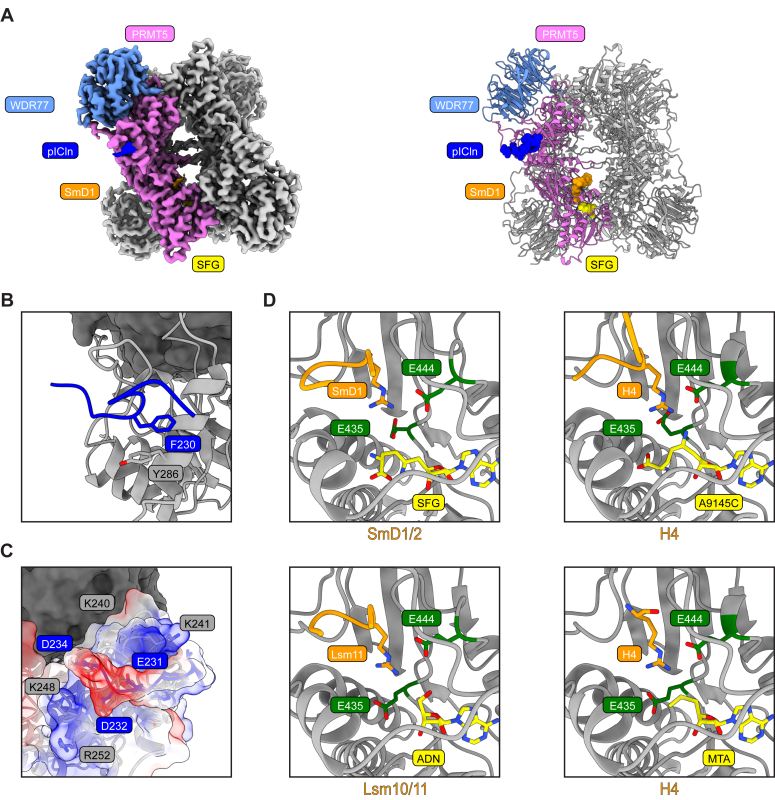
**Cryo-EM structure of PRMT5/WDR77/6S.***A*, cryo-EM map of the PRMT5/WDR77/6S complex (*left*) and model of the PRMT5/WDR77/6S complex (*right*). pICln PBM peptide is colored in *blue*, SmD1 GRG peptide in *orange*, and sinefungin in *yellow*. *B*, view of the pICln PBM peptide colored in *blue* illustrating the pi-pi stacking interaction with Y286 of PRMT5. *C*, electrostatic surface coloring showing charge complementarity between the positively charged PBM groove on PRMT5 and the negatively charged pICln PBM peptide. *D*, comparison of the active site of PRMT5 with SmD1/2 and sinefungin (*top left*), H4 with A9145C (*top right*, PDB: 4GQB), Lsm10/11 with adenosine (*bottom left*, PDB: 8G1U), and H4 with MTA (*bottom right*, PDB: 5FA5). Substrate is highlighted in *orange*, SAM analog highlighted in *yellow*, and the double E loop (E435 and E444) highlighted in *green*. cryo-EM, cryogenic electron microscopy; MTA, methylthioadenosine; PBM, PRMT5 binding motif; PRMT, protein arginine methyltransferase; SAM, S-Adenosyl-L-methionine.

Having determined the role of the PBM and GRG peptide motifs in substrate adaptor and substrate recruitment, we sought to structurally characterize a full-length PRMT5–adaptor–substrate complex. Previous studies had focused on determining the structure of the PBM peptide bound to PRMT5-WDR77 (23). Since our biochemical data suggests that substrates are anchored through the PBM peptide and the GRG motif, we asked whether the PRMT5–adaptor–substrate complex would assume a defined conformation. To test this, we reconstituted a PRMT5/WDR77/6S complex as previously described (Fig. S1*A*) (30). The 6S is an established structural complex of the Sm proteins comprised of pICln, SmD1, SmD2, SmE, SmF, and SmG, where pICln acts as an assembly chaperone of snRNP assembly (33). We collected cryo-EM data of this recombinant PRMT5/WDR77/6S complex in presence of the PRMT5 inhibitor sinefungin. A consensus reconstruction was refined to a resolution of 3 Å allowing us to build an atomic model. We observed clear density for the pICln PBM peptide and SmD1 GRG peptide, in accordance with a recently reported PRMT5/WDR77/U7 6S study (Fig. S2, *E* and *F*) (42). We also observed density for sinefungin (Fig. S2*G*). While the amino group of sinefungin is positioned differently than that of dehydrosinefungin (A9145C), this may be attributed to differences in structural determination, where, compared to X-ray crystallography, cryo-EM can sample different native conformational states of SAM analogs bound to PRMT5. The globular domains of the 6S complex were largely absent from 2D class averages and 3D reconstructions, owing to the flexible linkers attaching the 6S complex to the PBM and GRG peptide motifs (Figs 2*A* and S1*B*).

Our structural analysis revealed that the pICln PBM wedged in the previously described N-terminal groove on the TIM barrel of PRMT5 (23). The structure shows that binding of the pICln PBM to PRMT5 is driven by several types of interaction - the phenylalanine of the pICln PBM forms a pi-pi stacking interaction with Y286 of PRMT5 (Fig. 2*B*). There also exists significant charge complementarity between the positively charged PRMT5 PBM binding groove and the negatively charged pICln PBM peptide (Fig. 2*C*).

We were further able to build a six amino acid stretch of GRGRGR for the C-terminal tail of SmD1. Comparison of SmD1/2 with H4 and Lsm10/11 reveals a similar binding modality, where the target arginine is flanked by two glycines that enable flexibility to fit into the PRMT5 active site (Fig. 2*D*). The active site structure with SmD1 further elucidates how the structure of the methyl donor SAM cofactor plays a role in PRMT5 positioning for methylation. We see that with SmD1/2 with sinefungin and H4 with A9145C, the two glutamates of the double E loop (E435 and E444) that are critical for coordinating the target arginine for substrate methylation are poised to interact with the substrate. In structures of Lsm10/11 with adenosine and H4 with MTA, E435 is not positioned to interact with the target arginine. Inspection of the chemical structures of these SAM analogs suggests that their ornithine tails help push the side chain of E435 to interact with the substrate. Adenosine and MTA lack this terminal acid tail, thus providing a structural basis for how SAM positions the double E loop to catalyze substrate methylation.

This structural snapshot of the PRMT5/WDR77/6S complex further demonstrates the importance of the PBM peptide motif in regulating substrate adaptor and substrate recruitment to the PRMT5 methylosome and underscores how SAM structurally positions the active site to facilitate substrate methylation.

### PBM peptide binding does not allosterically activate PRMT5

**Figure 3:**
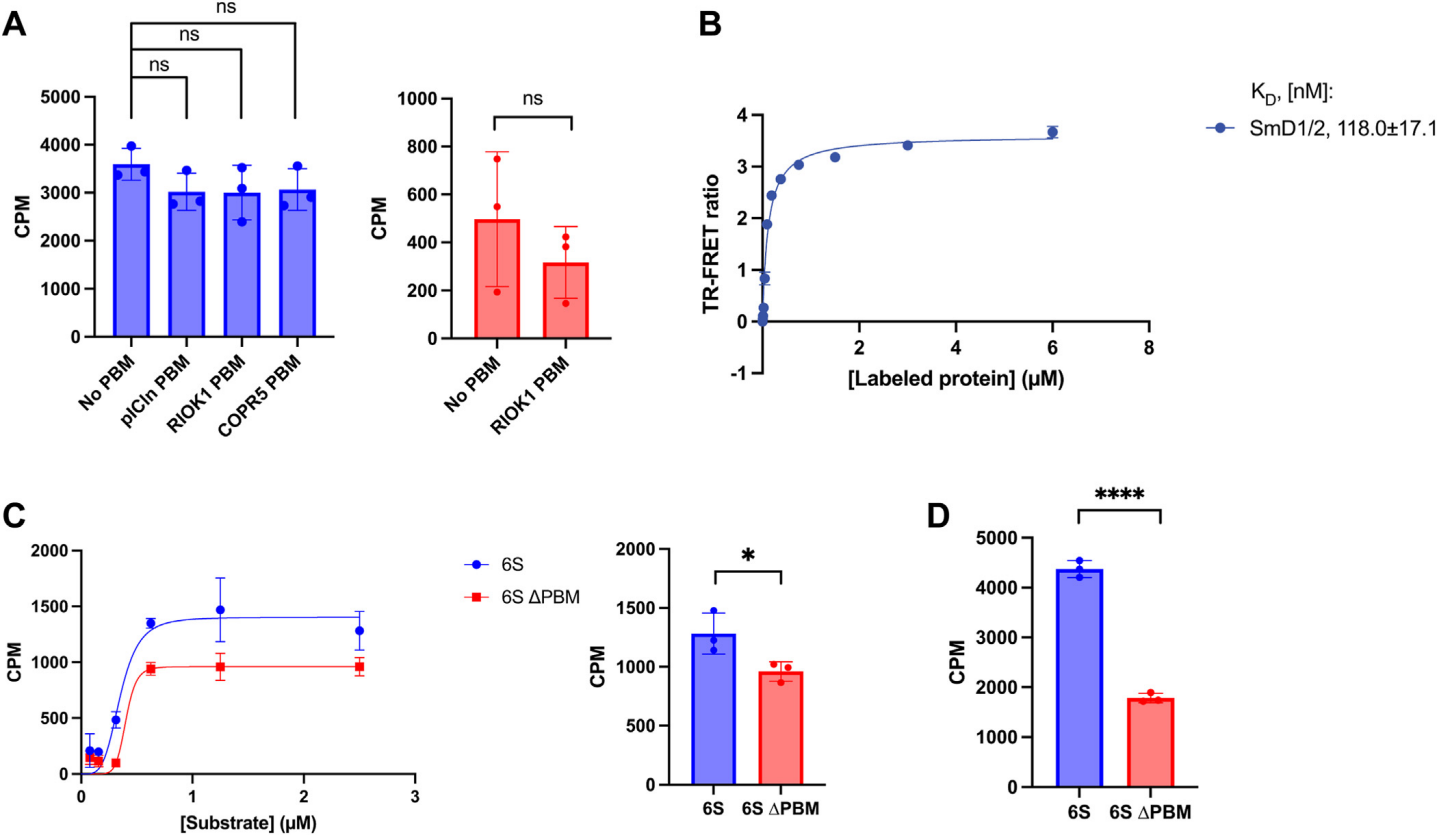
**pICln fine-tunes 6S methylation.***A*, SPA methylation of histone H4 (1-20) (*left*) and RPS10 (*right*). Incubation of PRMT5/WDR77 with PBM peptides does not increase methylation for histone H4 (1-20) or RPS10. Reactions were conducted in technical triplicates. *p* values calculated for H4 methylation with no PBM *versus* pICln, RIOK1, or COPR5 PBM peptide were 0.1221, 0.1948, and 0.1698, respectively. *p* value calculated for RPS10 methylation between no PBM and RIOK1 PBM was 0.3829. *B*, TR-FRET–based binding assay. SmD1/2 labeled by BODIPY-NHS ester was titrated into PRMT5/Flag-tagged WDR77 complex incubated with Tb-Flag M2 antibody. The *K*_D_ for SmD1/2 was determined to be 118.0 ± 17.1 nM. Data is represented as mean ± SD from three technical replicates. *C*, multiple turnover methylation assay. *Left*: SPA methylation was used to determine the kinetic parameters of full-length 6S and ΔPBM 6S. V_max_ for full-length 6S and ΔPBM 6S were 1402 ± 142 and 960.5 ± 74.6. K_half_ for full-length 6S and ΔPBM 6S were 352.4 ± 57.5 and 401.6 ± 113.3 nM. *Right:* comparison of full-length and ΔPBM 6S at 2.5 μM substrate concentration, with *p* value of 0.045. Data is represented as mean ± SD from three technical replicates. *D*, single turnover methylation assay. SPA methylation was measured on equimolar amounts of substrate with PRMT5/WDR77 (*p* value < 0.0001). Data is represented as mean ± SD from three technical replicates. All *p* values were calculated using an unpaired *t* test using GraphPad Prism 10 (https://www.graphpad.com/). PBM, PRMT5 binding motif; PRMT, protein arginine methyltransferase; SPA, scintillation proximity assay; TR-FRET, time-resolved FRET.

pICln binding to PRMT5 through its PBM peptide has been shown to promote methylation of Sm proteins in cells (23). Given the ability of PRMT5 to methylate SmD1/2 in absence of adaptor (Fig. 1*D*), we hypothesized that binding of the adaptor through its PBM peptide may allosterically activate PRMT5 methyltransferase activity, resulting in increased substrate methylation. To test this, we incubated PRMT5 with different PBM containing peptides and measured methylation of the histone H4 (1–20) peptide as a readout of methyltransferase activity. We observed no significant increase in activity when PRMT5 was incubated with different PBM peptides derived from pICIn or RIOK1 (Fig. 3*A*). Moreover, there was no specific substrate adaptor-substrate increase in activity when measuring RPS10 methylation after incubating PRMT5 with the RIOK1 peptide (Fig. 3*A*). To confirm that the PBM peptides were binding to PRMT5 under these assay conditions, we used a TR-FRET displacement assay with BODIPY-pICln as a tracer and saw all three PBM peptides were able to displace pICln at relevant concentrations (Fig. S3). These results demonstrate that binding of the isolated PBM peptide to the PRMT5 methylosome does not allosterically activate methyltransferase activity of PRMT5 towards substrates.

### Substrate adaptors facilitate substrate recruitment

While GRG containing proteins and peptides are methylated *in vitro*, we sought to test whether substrate adaptors would contribute to recruitment of substrates and thereby activity. To examine the binding affinity of GRG-containing substrates with PRMT5/WDR77, we measured the affinity between PRMT5/WDR77 and the substrate SmD1/2 and observed a *K*_D_ of 118.0 ± 17.1 nM (Fig. 3*B*). This suggested that *in vitro* the pICln substrate adaptor is not necessary for substrate binding to the PRMT5 methylosome, consistent with the ability of PRMT5 to methylate SmD1/2 in the absence of pICln (Fig. 1*D*). Using SPA, we determined the kinetic parameters for methylation of full-length and ΔPBM 6S complexes in a multiple turnover enzymatic regime. The full-length 6S complex exhibited a V_max_ of 1402 ± 142 and a K_half_ of 352.4 ± 57.5 nM, while ΔPBM 6S exhibited a V_max_ of 960.5 ± 74.6 and a K_half_ of 401.6 ± 113.3 nM (Fig. 3*C*). These differences in kinetic parameters suggest that although a substrate adaptor is not required for substrate methylation, it does fine-tune methylation levels and enzyme kinetics. Given that the isolated SmD1/2 was able to bind to PRMT5 alone, we further tested methylation in a single turnover enzymatic regime to restrict active site saturation by SmD1. We observed a more profound difference in methylation between full-length and ΔPBM 6S complex, emphasizing the role of substrate adaptor binding to PRMT5 in the context of promoting substrate methylation (Fig. 3*D*). Interestingly, the transplant of the pICln PBM tail to different components of the 6S complex did not rescue methylation (Fig. S4), suggesting an orientation-specific effect on methylation and not purely avidity provided by the second binding site. Collectively, these studies clarify that although not required for PRMT5 substrate binding and methylation *in vitro*, adaptor proteins are involved in promoting specific PRMT5 activity.

## Discussion

PRMT5 is responsible for methylating a diverse set of critical substrates related to gene expression and proliferation and represents an important therapeutic target for the treatment of cancer. It has been shown that substrate adaptors such as RIOK1 and pICln contribute to the activity and specificity of PRMT5. Here, we provide mechanistic details on how different substrate adaptors compete for access to PRMT5 and facilitate the methylation of distinct substrates. Substrate adaptors compete for binding to PRMT5 through their PBM peptides, and display subtle difference in binding affinity, which could contribute to differential activity. While the position of the PBM peptide on the substrate adaptor/substrate complex is important, it does not lead to a highly structured PRMT5–substrate complex but rather substrate adaptors lead to more efficient recruitment and methylation of the substrate by proximity. We also find that the canonical methylation motif GRG is placed in the active site of PRMT5, providing a secondary binding site. In fact, substrates containing the GRG motif are methylated independently of substrate adaptors albeit at lower rates. Overall, we demonstrate that the PBM serves as the main attachment point between substrate adaptor and PRMT5 and provide a structural framework for the consensus GRG motif that PRMT5 preferentially methylates.

**Figure 4:**
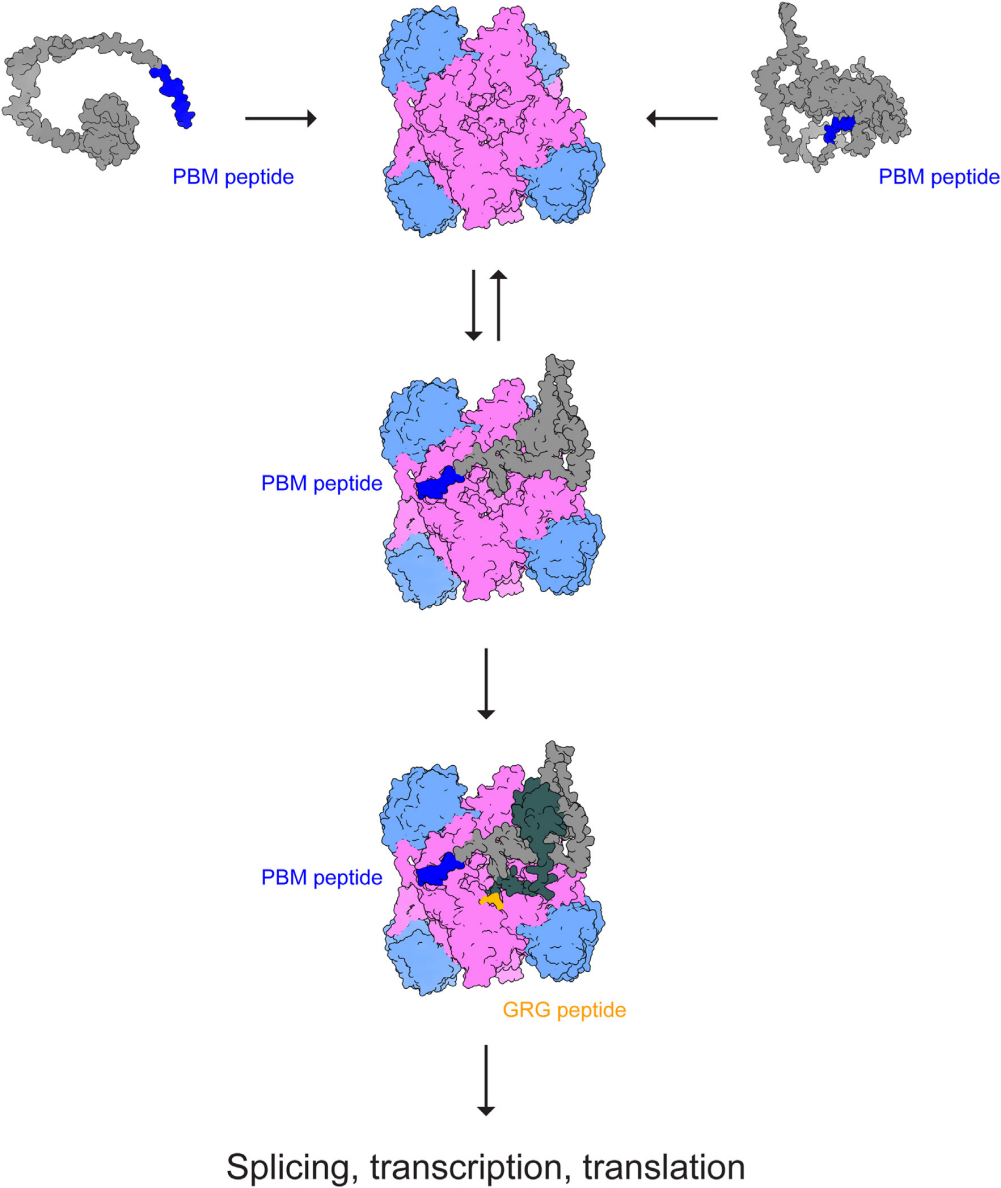
**The PRMT5 methylosome as a modular peptide binding platform.** Schematic of substrate adaptor and substrate recruitment to PRMT5. Substrate adaptors compete for binding to the PRMT5 methylosome through their PBM peptide. By serving as an extra attachment point through the PBM peptide, substrate adaptors subsequently serve to recruit substrates to the PRMT5 methylosome to increase methylation on the substrate GRG peptide. PBM, PRMT5 binding motif; PRMT, protein arginine methyltransferase.

We also found evidence that, instead of an all-or-nothing event, at least *in vitro*, substrate adaptors serve to finely modulate substrate methylation as flexible tethering modules (Fig. 4). Unlike other systems, such as the cullin-RING E3 ligase system, where substrate adaptors are necessary for *in vitro* binding and activity, PRMT5 already has a basal level of affinity and activity against substrates containing GRG motifs in the absence of adaptor protein. Furthermore, adaptors may have additional roles in a crowded cellular environment, such as to localize the PRMT5 methylosome to specific substrates and enhance their methylation. For example, COPR5, another PBM-containing adaptor, has been reported to mediate nuclear localization of PRMT5 to activate histone methylation (20). Other mechanisms of regulating PRMT5 substrate specificity, such as differential transcription or translation of GRG-containing proteins, may also help alter protein methylation.

PRMT5/WDR77 has been shown to form a complex with nucleoplasmin, and thus it remains to be seen whether PRMT5/WDR77 contains additional peptide binding regions that enable the PRMT5 methylosome to engage other types of interacting partners (22). Nonetheless, multiple structures, including the one we describe here, suggest that PRMT5 contains two main hot spots for peptide binding: the TIM barrel, which mediates interactions with adaptors, and the active site, which accommodates substrate interactions. Future studies will be needed to examine existence of additional binding interfaces and their functional relevance.

In conclusion, our work establishes the structural and biochemical foundation for how adaptors interact with PRMT5 through peptide motifs to control methylation and establishes a general framework for the biochemical basis of PRMT5 interactors.

## Experimental procedures

### Cloning, protein expression, and purification

PRMT5/WDR77, RIOK1, and RPS10 variants were purified from Trichoplusia Ni following affinity, ion exchange, and size-exclusion chromatography. pICln, SmD1/2, and SmE/F/G variants were purified from LBSTR *Escherichia coli* strains (Kerafast, EC1002) following a similar protocol. Detailed purification methods can be found in Supplemental Information.

### TR-FRET assays

TR-FRET–based binding assays were conducted with PRMT5/biotinylated WDR77 and terbium-coupled streptavidin or PRMT5/Flag-WDR77 and Tb-Flag-M2 antibody, with BODIPY-labeled interactors (43). Displacement assays were conducted by titrating in unlabeled interactors. Detailed methods on TR-FRET binding and displacement assays can be found in Supplemental Information.

### PRMT5/WDR77 methyltransferase assays

PRMT5/WDR77-based methylation assays were monitored either with specific antibodies against mono methyl-arginine or SDMA or as a SPA using H^3^-SAM. Information on 6S substrate reconstitution and methylation assays is detailed in Supplemental Information.

### Cryo-EM data processing and model building

Cryo-EM sample preparation and data collection details are available in Supplemental Information. All processing was performed in cryoSPARC v4.4.1 (https://cryosparc.com/) and RELION5.0-beta (https://relion.readthedocs.io/en/release-5.0/) (44, 45). Resolutions given are based on the Fourier shell correlation 0.143 threshold criterion (46, 47). 3615 movies were corrected for beam-induced motion and contrast transfer function was estimated on-the-fly in cryoSPARC live. 3,351,462 particles were picked after template picking. A consensus reconstruction from 1,006,290 particles at 3.1 Å was used as a seed model to classify 2,045,410 particles from TOPAZ particle picking. After four rounds of heterogeneous refinement, 377,557 particles resulted in a 2.92 Å consensus refinement. These 377,557 particles further underwent D2 symmetry expansion and were imported into RELION. 3D classification without alignment was performed using soft masks around the PBM peptide and PRMT5 active site. Particles belonging to classes with the most pronounced density for the pICln PBM or SmD1 RG tail were imported back into cryoSPARC and local refinement focused on the respective areas resulted in reconstructions at 3.38 Å and 3.18 Å for PRMT5-PBM (from 285,661 particles) and for PRMT5-GRG (from 530,949), respectively. More details on data collection parameters, model building, and refinement statistics are available in Supplemental Information.

## Data availability

Cryo-EM maps and coordinates of the PRMT5/WDR77/6S consensus refinement, PBM local refinement, and GRG local refinement are available in the EMDB and PDB under accession codes EMD-47477 (PRMT5/WDR77/6S consensus, PDB: 9E3B), EMD-47476 (PRMT5/WDR77/PBM local refine, PDB: 9E3A), and EMD-47478 (PRMT5/WDR77/GRG local refine, PDB: 9E3C). The data in this study are available upon request.

## Conflict of interest

E. S. F. is a founder, scientific advisory board (SAB) member, and equity holder of Civetta Therapeutics, Proximity Therapeutics, Stelexis Biosciences, and Neomorph, Inc. (also board of directors). He is an equity holder and SAB member for Avilar Therapeutics, Photys Therapeutics, and Ajax Therapeutics and an equity holder in Lighthorse Therapeutics, CPD4 and Anvia Therapeutics. E. S. F. is a consultant to Novartis, EcoR1 capital, Odyssey, and Deerfield. The Fischer lab receives or has received research funding from Deerfield, 10.13039/100004336Novartis, Ajax, Interline, 10.13039/100004326Bayer, and Astellas.

W. R. S. is a Board or SAB member and/or holds equity in CJ Bioscience, Delphia Therapeutics, Ideaya Biosciences, Pierre Fabre, Red Ridge Bio, Scorpion Therapeutics, and has consulted for Array, Astex, Epidarex Capital, Ipsen, Merck Pharmaceuticals, Sanofi, Servier and Syndax Pharmaceuticals and receives research funding from Bayer Pharmaceuticals, 10.13039/100002491Bristol-Myers Squibb, Boehringer-Ingelheim, Ideaya Biosciences, Calico Biosciences, and Servier Pharmaceuticals.

The other authors declare that they have no conflicts of interest with the contents of this article.

## Supporting information

Supplemental Information

Supporting Table1
