# Supplementary material for: Substrate adaptors are flexible tethering modules that enhance substrate methylation by the arginine methyltransferase PRMT5
Source: J Biol Chem. 2025 Jan 8;301(2):108165. doi: 10.1016/j.jbc.2025.108165 (PMC11847536; doi:10.1016/j.jbc.2025.108165)
Supplement: Supplemental Information [file mmc1.docx]

**Supplemental Information**

Cloning, protein expression, and purification

PRMT5 (Uniprot: O14744) , WDR77 (Uniprot: Q9BQA1), RIOK1 (Uniprot: Q9BRS2), RIOK1∆PBM (24-568), and RPS10 (Uniprot: P46783) were synthesized as double-stranded DNA (gBlocks, IDT) and inserted into pAC8-derived vectors using Gibson assembly (NEB, E2621S)(1). Baculovirus were generated and amplified in Spodoptera frugiperda SF9 cells grown in ESF921 medium (Expression Systems). Trichoplusia Ni High Five cells (Thermo Fisher Scientific) at 2 x 10^6^ cells/mL were infected with 1.5% untagged PRMT5 and tagged WDR77 baculovirus,1.5% Flag-RIOK1 baculovirus, or 1.5% Strep-Avi-RPS10 baculovirus, and incubated for 40 hours. High Five cells were pelleted, resuspended in lysis buffer (50 mM HEPES/KOH pH 8.0, 200 mM NaCl) supplemented with protease inhibitors, lysed via sonication, and cleared with ultracentrifugation (60 min, 186,000g). The soluble fraction was passed over Strep-Tactin XT (IBA) or Flag resin (GenScript), washed with lysis buffer and eluted with buffer supplemented with 50 mM biotin (MCE) or 0.2 mg/mL 1X FLAG (DYKDDDK) peptide. Proteins were purified by ion exchange chromatography on a Poros 50HQ or 50HS. Samples were then polished with a Superose6Increase column (GE Healthcare) in SEC1 buffer (30 mM HEPESH/KOH pH 7.4, 150 mM NaCl, 3 mM TCEP, supplemented with 10% glycerol for methylation assay variants) for PRMT5/WDR77 variants or a Superdex75 (GE Healthcare) for RIOK1 and RPS10. Relevant fractions were pooled, concentrated using ultrafiltration (Millipore), and flash frozen in liquid nitrogen. For cryo-EM, PRMT5/Strep-WDR77 was TEV cleaved overnight after ion exchange chromatography with TEV. PRMT5/Strep-Avi-WDR77 and Strep-Avi-RPS10 were biotinylated overnight after ion exchange chromatography as described previously(2).

Full-length (Uniprot: P54105) and ∆PBM pICln (1-224) were subcloned into a pNIC-Bio2 (SGC Oxford) plasmid with an N-terminal 10X His tag. SmD1/2 (Uniprot: P62314, P63216) and SmE/F (Uniprot: P62304, P62306) were synthesized as double-stranded DNA and inserted into a pET-DUET expression plasmid with an N-terminal Strep tag or 6X His tag using Gibson assembly. SmE, SmF, or SmG pICln fusions were ordered as double-stranded DNA with the C-terminal pICln tail (136-237) appended to their C-terminus. Plasmids were mutagenized by linearization with primers containing sequences for the desired mutation, DpnI treatment (NEB, R0176S), and polynucleotide kinase (NEB, M0201S) and T4 DNA ligase (NEB, M0202S) treatment for ligation. SmG (Uniprot: P62308) was synthesized as double-stranded DNA and inserted into a pCDF tagless expression plasmid using Gibson assembly. LBSTR *E. coli* strains (Kerafast, EC1002) were transformed and grown at 37 °C until an optical density at 600 nm of 0.6-0.8. Protein expression was induced at 37 °C for 3 hours or 18 °C overnight using 0.4 – 1 mM IPTG. Samples were processed similarly, except for the addition of 20 mM imidazole in the lysis buffer for His variants. His variants were eluted from nickel resin with a 150-750 mM imidazole gradient. Proteins were subsequently purified by ion exchange chromatography with a Poros 50HQ or 50HS. Proteins were then purified on a Superdex75 size exclusion column with SEC2 buffer (30 mM HEPES pH 7.4, 200 mM NaCl, 2 mM TCEP). For cryo-EM and 6S complex formation, all His tagged pICln and SmE/F/G variants were TEV cleaved overnight after ion exchange. Prior to size exclusion, cleaved variants were flowed through nickel resin. For cryo-EM, Strep-SmD1/2 was TEV cleaved after size exclusion. For SPA, Strep-Avi-SmD1/2 full length and ∆GRG variants were biotinylated overnight after biotin elution or during expression through co-expression with BirA and 100 µM biotin in the culture media.

6S complex formation

TEV-cleaved PRMT5/WDR77 was mixed with an 8-fold molar excess of TEV-cleaved pICln, TEV-cleaved SmD1/2, and TEV-cleaved SmE/F/G with 1 M NaCl. The complex was dialyzed overnight against 30 mM HEPES pH 7.4, 150 mM NaCl, 3 mM TCEP. The following day, the complex was purified on a Superose6Increase column in 30 mM HEPES pH 7.4, 150 mM NaCl, 3 mM TCEP, 122.14 µM sinefungin (MedChemExpress, HY-101938) and the relevant fractions containing PRMT5/WDR77/6S were pooled, concentrated, and plunged.

For SPA, 6S complex variants were reconstituted by adding TEV-cleaved pICln, TEV-cleaved SmE/F/G, variants, and biotinylated SmD1/2 variants in a 1:1:1 molar ratio with 1 M NaCl, Complexes were dialyzed overnight against 30 mM HEPES pH 7.4, 150 mM NaCl, 2 mM TCEP. The complexes were purified the following day on a Superdex200Increase column, and the relevant fractions were pooled, concentrated using ultrafiltration, and flash frozen in liquid nitrogen.

Fluorescent protein labeling

His-pICln, His-pICln ∆PBM, Flag-RIOK1, and Flag-RIOK1 ∆PBM were incubated with a 2-fold molar excess of BODIPY-Maleimide (Thermo Fisher Scientific, B10250) for 60 minutes at room temperature. Labeling reactions were quenched with 10 mM β-mercaptoethanol and buffer exchanged into 30 mM HEPES pH 7.4, 150 mM NaCl, 2 mM TCEP with Zeba Spin Desalting Columns (Thermo Fisher Scientific, 89882). A_280_ and A_503_ were calculated for each sample and degree of labeling (DOL) was calculated by measuring the relative concentration of BODIPY using an extinction coefficient of 80,000 M^-1^cm^-1^. The DOL of pICln, pICln ∆PBM, RIOK, and RIOK1 ∆PBM was calculated to be 0.93, 0.97, 0.68, 0.85, respectively.

Strep-SmD1/2 was incubated with a 2-fold molar excess of BODIPY-Nhs. Labeling reaction was quenched with 100 mM Tris pH 8.0 after 1 hour at room temperature, and buffer exchanged. The DOL of SmD1/2 was 0.69.

TR-FRET assays

For equilibrium binding assays, 2 or 10 nM PRMT5/biotinylated WDR77 was incubated with 2 nM terbium-coupled streptavidin. PRMT5/biotinylated WDR77 was mixed with 0.0049 – 5 µM BODIPY-labeled proteins buffered with 25 mM HEPES/KOH pH 7.4, 50 mM NaCl, 1% bovine serum albumin, 0.05% Tween-20, 2 mM TCEP in a 384-well microplate (Corning, 4514) for 60 minutes at room temperature. Total reaction volume was 15 µL. Plates were read on a PHERAstar FX (BMG Labtech) plate reader. Emission at 490 nm for terbium and 520 nm for BODIPY were recorded after terbium excitation at 337 nM with a 70 µs delay over 200 µs. Background signal was calculated by incubating the same concentration range of BODIPY-labeled proteins with 2 nM terbium-coupled streptavidin, which was subtracted from the total signal. Equilibrium binding constants were calculated by averaging 5 cycles and fitting the 520/490 TR-FRET ratio with the one-site (specific binding) equation in GraphPad Prism.

For equilibrium binding assays with SmD1/2, 10 nM PRMT5/Flag-WDR77 was incubated with 8 nM Tb-FLAG-M2 antibody. PRMT5/Flag-WDR77 was mixed with 0.0049 – 5 µM BODIPY-labeled SmD1/2 buffered with 25 mM HEPES/KOH pH 7.4, 50 mM NaCl, 1% BSA, 0.05% Tween-20, and 0.075 mM TCEP.

Displacement assays were conducted with 10 nM PRMT5/biotinylated WDR77, 2 nM terbium-coupled streptavidin, and 39 nM BODIPY-labeled pICln. Unlabeled proteins were titrated over a 0.000007 – 30 µM or 0.00001 – 50 µM range for PBM peptides. The 520/490 TR-FRET ratio was averaged over 5 cycles and analyzed with the [inhibitor] vs. response – variable slope (four parameters) equation on GraphPad Prism.

Methylation western blot assay

5 µM SmD1/2 or SmD1∆GRG/SmD2 was incubated with 100 nM PRMT5/Flag-WDR77 and 20 µM S-Adenosyl-L-Methionine (Cayman Chemical, 13956) buffered with 50 mM HEPES/KOH pH 7.2, 50 mM NaCl, 2 mM TCEP for 150 minutes at 37°C. Western blots were transferred using the iBlot 2 dry blotting system (Thermo Fisher Scientific, IB21001) and visualized on a LI-COR Odyssey CLx. Antibodies used were MMA (Cell Signaling Technology, 8015), SDMA (Cell Signaling Technology, 13222), and donkey anti-rabbit secondary (LI-COR, 926-32213) antibodies. The same amount of sample was loaded onto a 4-20% TGX gel (Bio-Rad, 4561096) as a loading control and imaged on a Gel Doc XR+ System (BioRad) after staining with Coomassie G-250 (Thermo Fisher Scientific). MMA and SDMA antibody specificity was verified in biochemical assays by PRMT5 methylation.

SPA assay

For SmD1/2, histone H4, and RPS10 methylation assays, 1 µM substrate was incubated with 100 nM PRMT5/Flag-WDR77 and 5 µM H^3^-S-Adenosyl-L-Methionine/10 µM S-Adenosyl-L-Methionine (Revvity, NET155V001MC) buffered with 50 mM HEPES/KOH pH 7.2, 50 mM NaCl, 2 mM TCEP at room temperature for 10 minutes in 7 µL total volume. For reactions with the PBM peptide, 20 µM of peptide was used. Reactions were quenched with 1.4 µL 10% trifluoroacetic acid (Millipore Sigma, T6508). Quenched reactions were diluted with SEC2 buffer prior to adding SPA beads (Revvity, RPNQ0006). Reaction mixes were added to a 96-well plate (Revvity, 6055290) and read on a MicroBeta2 plate reader (Perkin Elmer) over 30 cycles. Background signal was calculated by the signal of SPA beads with the hot/cold SAM mix and was subtracted from each measurement. Final SPA bead concentration was 1.96 mg/mL. Reactions were averaged over 5 cycles.

For 6S multiple turnover methylation assays, 10 nM PRMT5/Flag-WDR77 was incubated with 6S complexes at a range of 0.078 – 2.5 µM. Reactions were conducted for 48 minutes at room temperature. Final SPA bead concentration was 5.89 mg/mL. Data was analyzed with the allosteric sigmoidal equation on GraphPad Prism.

For 6S single turnover methylation assays, 500 nM PRMT5/Flag-WDR77 was incubated with 500 nM substrate, and reactions were run for 10 minutes. Final SPA bead concentration was 1.97 mg/mL.

Peptide synthesis

All peptides were ordered as synthetic peptides from GenScript. pICln PBM: TVAGQFEDADVDH. RIOK1 PBM: VVPGQFDDADSSD. COPR5 PBM: FETGQFDDAED. C-terminally biotinylated histone H4 (1-20): SGRGKGGKGLGKGGAKRHRK

EM sample preparation and data collection

1.25 mg/mL PRMT5/WDR77/6S after size exclusion chromatography was incubated with 3.33 mM sinefungin for 30 minutes on ice prior to plunging. Grids were prepared with a Leica EM-GP at 10 °C at 90% relative humidity. 4 µL were applied to glow-discharged Quantifoil 1.2/1.3 300 grids and, after a 10 second pre-blot time, excess sample was blotted away for 3 seconds followed by a 4 second post-blot time prior to vitrification. Grids were imaged on a Talos Arctica equipped with a Gatan K3 detector. Using SerialEM (4.1.0beta), 3,615 movies were acquired at 200 kV at 36,000x magnification in counting mode with a pixel size of 1.1 Å/pixel. Movies were recorded with 25 holes per stage position at a defocus range of -0.8 - -2.2 µm, exposure time of 5 s, and total exposure dose of 53.112 e^-^/Å^2^.

Model building

PRMT5/WDR77/6S consensus model and PRMT5/WDR77/PBM locally refined model were constructed by rigid-body fitting the PRMT5/WDR77/pICln PBM peptide crystal structure (6V0O) into the cryo-EM density in ChimeraX, relaxed into the density with ISOLDE, and manually adjusted in COOT(3-6). The SmD1 C-terminal tail in the PRMT5 active site in the PRMT5/WDR77/6S consensus model and PRMT5/WDR77/GRG locally refined model was built de novo. All final maps were used for model building in COOT (v0.9.6)(4). All models were protonated (phenix.reduce) and refined against the main maps from cryoSPARC using phenix.real_space_refine (v.1.20.1-4487)(7, 8). Models were iteratively refined in ISOLDE (v1.3), phenix.real_space_refine, and manual inspection in COOT(4). Figures for maps and models were generated in UCSF ChimeraX(3). Structural biology applications used in this project were compiled and configured by SBGrid(9).

**References**

1. Abdulrahman, W., Uhring, M., Kolb-Cheynel, I., Garnier, J. M., Moras, D., Rochel, N. *et al.* (2009) A set of baculovirus transfer vectors for screening of affinity tags and parallel expression strategies Anal Biochem **385**, 383-385 10.1016/j.ab.2008.10.044

2. Cavadini, S., Fischer, E. S., Bunker, R. D., Potenza, A., Lingaraju, G. M., Goldie, K. N. *et al.* (2016) Cullin-RING ubiquitin E3 ligase regulation by the COP9 signalosome Nature **531**, 598-603 10.1038/nature17416

3. Goddard, T. D., Huang, C. C., Meng, E. C., Pettersen, E. F., Couch, G. S., Morris, J. H., andFerrin, T. E. (2018) UCSF ChimeraX: Meeting modern challenges in visualization and analysis Protein Sci **27**, 14-25 10.1002/pro.3235

4. Croll, T. I. (2018) ISOLDE: a physically realistic environment for model building into low-resolution electron-density maps Acta Crystallogr D Struct Biol **74**, 519-530 10.1107/S2059798318002425

5. Emsley, P., Lohkamp, B., Scott, W. G., andCowtan, K. (2010) Features and development of Coot Acta Crystallogr D Biol Crystallogr **66**, 486-501 10.1107/S0907444910007493

6. Mulvaney, K. M., Blomquist, C., Acharya, N., Li, R., Ranaghan, M. J., O'Keefe, M. *et al.* (2021) Molecular basis for substrate recruitment to the PRMT5 methylosome Mol Cell **81**, 3481-3495 e3487 10.1016/j.molcel.2021.07.019

7. Adams, P. D., Afonine, P. V., Bunkoczi, G., Chen, V. B., Davis, I. W., Echols, N. *et al.* (2010) PHENIX: a comprehensive Python-based system for macromolecular structure solution Acta Crystallogr D Biol Crystallogr **66**, 213-221 10.1107/S0907444909052925

8. Afonine, P. V., Poon, B. K., Read, R. J., Sobolev, O. V., Terwilliger, T. C., Urzhumtsev, A., andAdams, P. D. (2018) Real-space refinement in PHENIX for cryo-EM and crystallography Acta Crystallogr D Struct Biol **74**, 531-544 10.1107/S2059798318006551

9. Morin, A., Eisenbraun, B., Key, J., Sanschagrin, P. C., Timony, M. A., Ottaviano, M., andSliz, P. (2013) Collaboration gets the most out of software Elife **2**, e01456 10.7554/eLife.01456

**Supplemental Figure 1** **Cryo-EM processing workflow of PRMT5/WDR77/6S complex.** *A*, (Left) Representative fraction from the PRMT5/WDR77/6S peak from gel filtration of PRMT5/WDR77/6S complex reconstitution. (Right) Chromatogram of gel filtration of PRMT5/WDR77/6S complex reconstitution. Peaks are labeled representing the PRMT5/WDR77/6S complex, excess 6S complex, and excess sinefungin. *B*, Overview of processing workflow of PRMT5/WDR77/6S from raw micrograph to final maps. Steps outside the dotted box were performed in cryoSPARC and steps in the dotted box were performed in RELION. Particles belonging to the colored volumes were used in subsequent steps.

**Supplemental Figure 2 Map validation and quality for the PRMT5/WDR77/6S structure.** *A-C*, FSC plots (top) and viewing distribution plots (bottom) for all deposited maps ((A), EMD-47477, (B), EMD-47476, (C), EMD-47478). *D*, Final maps colored according to local resolution (left) and model-to-map FSC for deposited structures with values given for FSC(model)=0.5 (right) *E-H*, Density examples for the pICln PBM peptide (E), SmD1 substrate tail (F), sinefungin (G), and top view of WDR77 (H).

**Supplemental Figure 3** **PBM peptides bind to PRMT5/WDR77** *A*, TR-FRET displacement assay demonstrating the three PBM peptides’ ability to displace BODIPY-labeled pICln binding with PRMT5/WDR77. Data is represented as mean ± SD from 3 technical replicates.

**Supplemental Figure 4** **Substrate methylation depends on pICln linker orientation** *A,* SPA methylation suggesting that transplantation of the pICln PBM tail to different subunits of the 6S complex does not rescue SmD1 methylation. Data is represented as mean ± SD from 3 technical replicates.
