# Supplementary material for: Substrate adaptors are flexible tethering modules that enhance substrate methylation by the arginine methyltransferase PRMT5
Source: J Biol Chem. 2025 Jan 8;301(2):108165. doi: 10.1016/j.jbc.2025.108165 (PMC11847536; doi:10.1016/j.jbc.2025.108165)
Supplement: Supporting Table1 [file mmc2.docx]

Extended Data Table 1 | Data collection and refinement statistics

|  | PRMT5/WDR77/6S | | |
| --- | --- | --- | --- |
| Microscope | Thermo Fisher Scientific Talos Arctica | | |
| Voltage (kV) | 200 | | |
| Camera | Gatan K3 | | |
| Magnification | 36,000x | | |
| Pixel size (Å) | 1.1 | | |
| Total electron exposure (e^-^/Å^2)^ | 53.112 | | |
| Number of frames (no.) | 50 | | |
| Defocus range (µm) | -0.8 - -2.2 | | |
| Data collection software | SerialEM 4.1.0beta | | |
| Micrographs collected (no.) | 3,615 | | |
| Total extracted particles (no.) | 2,100,293 (TOPAZ) | | |
|  | Map 1 | Map 2 | Map 3 |
|  | Consensus | PBM | GRG |
| EMDB accession code | 9E3B | 9E3A | 9E3C |
| PDB accession code | EMD-47477 | EMD-47476 | EMD-47478 |
| Final particles used (no.) | 377,557 | 285,661 | 530,317 |
| Map resolution (Å) | 3.06 | 3.36 | 3.19 |
| FSC threshold | 0.143 | 0.143 | 0.143 |
| Model composition |  |  |  |
| Non-hydrogen atoms | 30,152 | 4,521 | 5,086 |
| Protein residues | 3,799 | 577 | 626 |
|  |  |  |  |
| *B* factors (Å^2^) |  |  |  |
| Protein | 114.4 | 110.5 | 110.3 |
| Water | - | - | - |
| R.m.s. deviations |  |  |  |
| Bond lengths (Å^2^) | 0.003 | 0.003 | 0.003 |
| Bond angles (°) | 0.520 | 0.589 | 0.558 |
| Validation |  |  |  |
| MolProbity Score | 1.54 | 2.04 | 1.27 |
| Clash score | 6.00 | 9.28 | 3.38 |
| Rotamer outliers (%) | 1.02 | 2.00 | 0.54 |
| Ramachandran plot |  |  |  |
| Favored (%) | 96.73 | 95.40 | 97.26 |
| Allowed (%) | 3.24 | 4.60 | 2.74 |
| Disallowed (%) | 0.03 | 0.00 | 0.00 |
