# Supplementary figures and images for: Substrate adaptors are flexible tethering modules that enhance substrate methylation by the arginine methyltransferase PRMT5
Source: J Biol Chem. 2025 Jan 8;301(2):108165. doi: 10.1016/j.jbc.2025.108165 (PMC11847536; doi:10.1016/j.jbc.2025.108165)

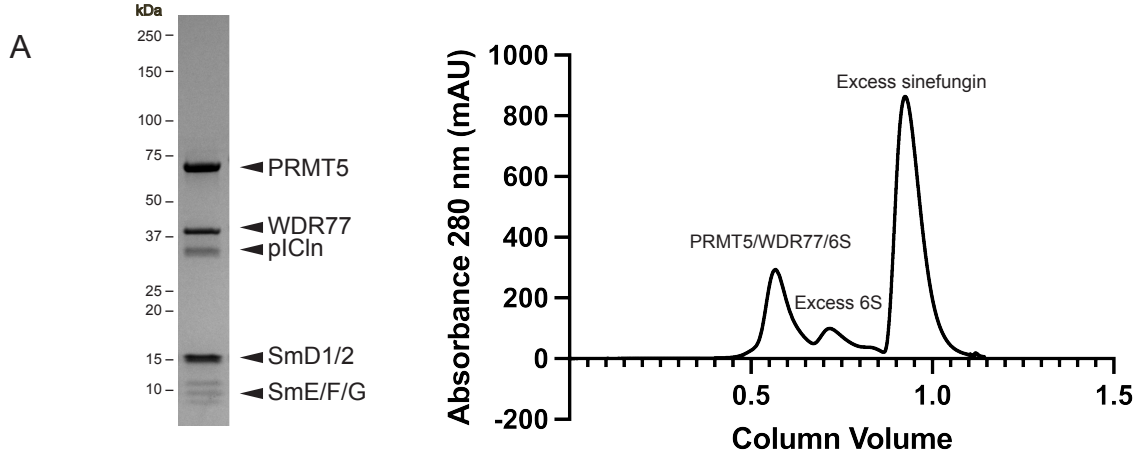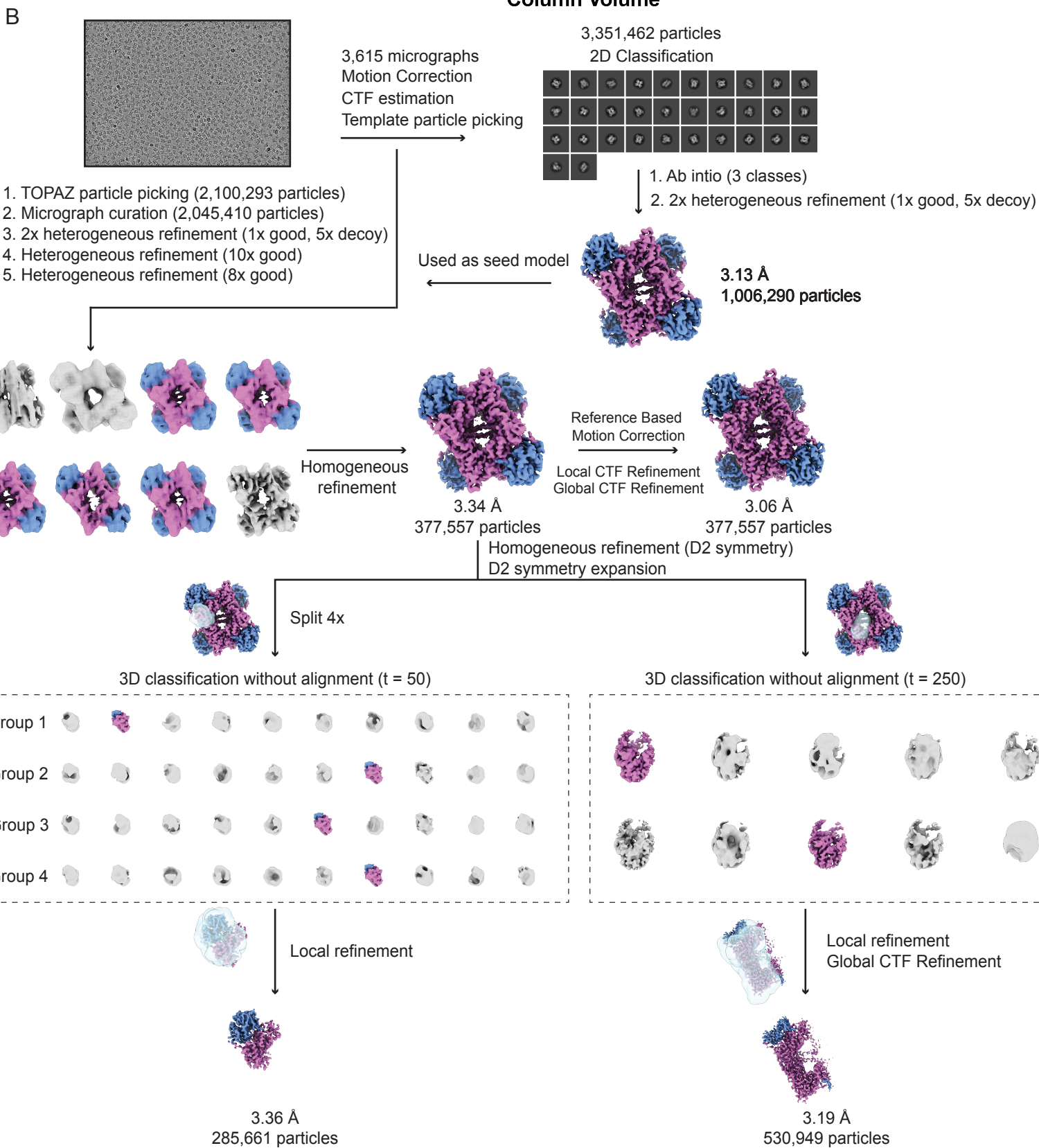

Supplement: Supplemental Figure 1 [file mmc3.pdf]

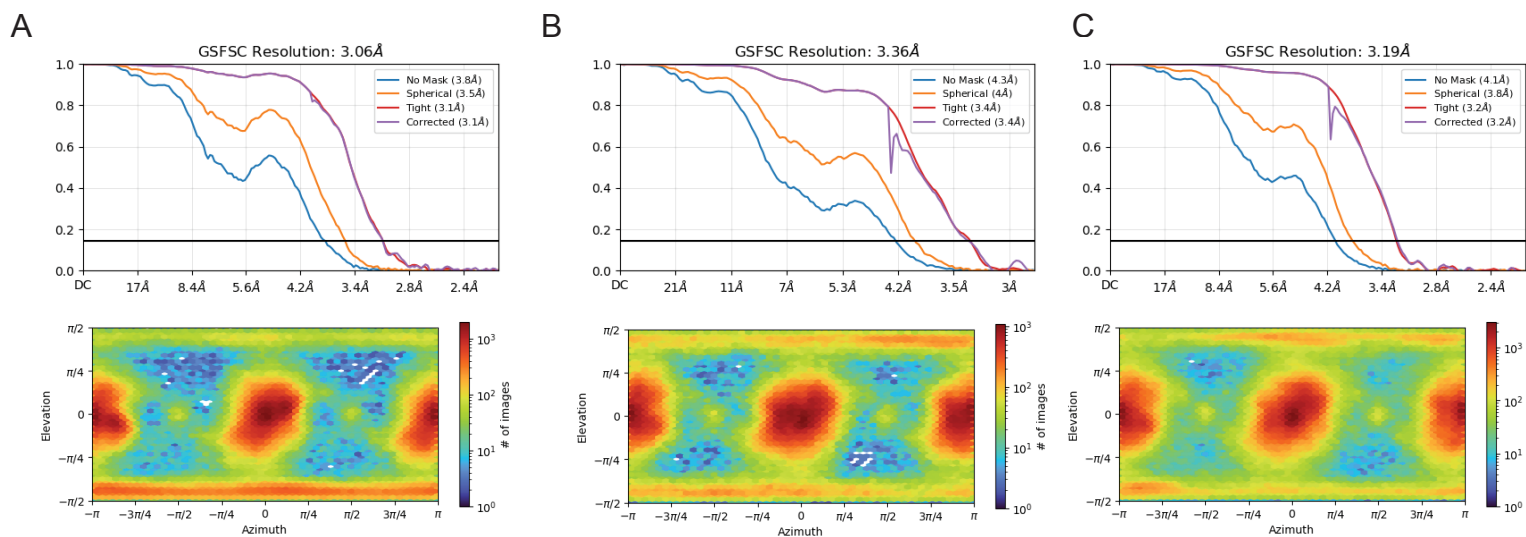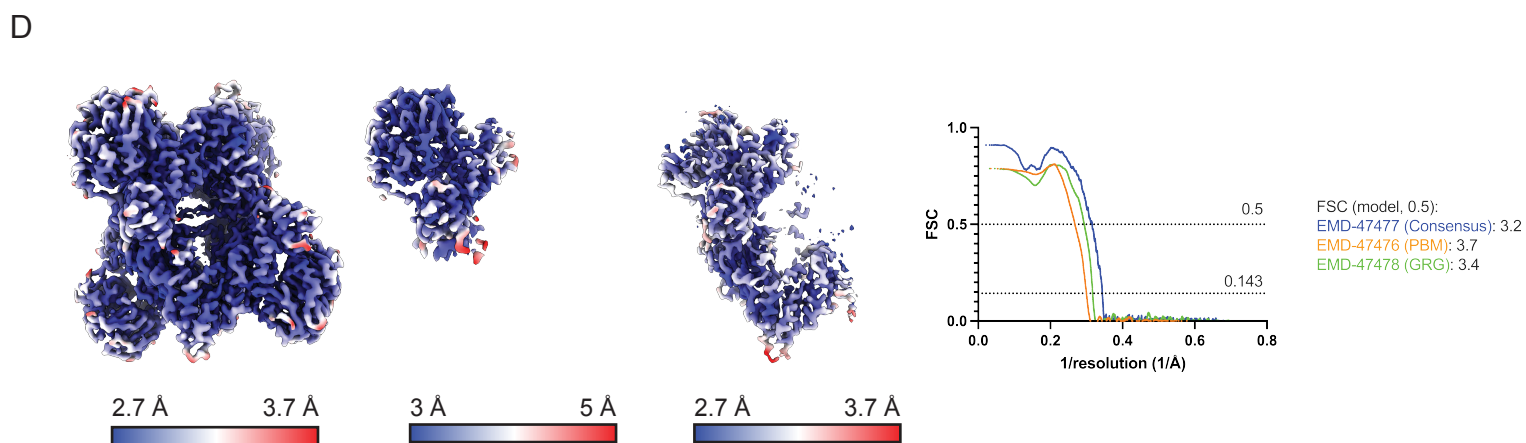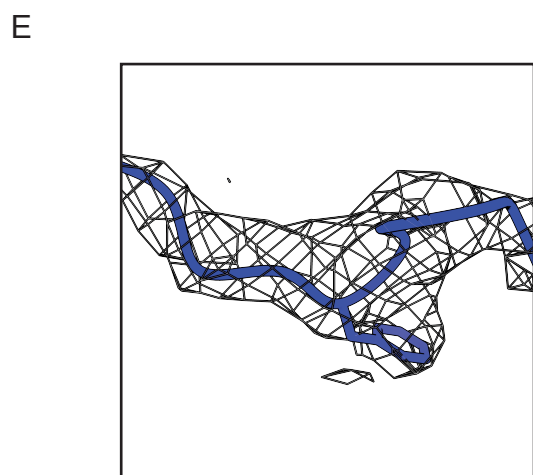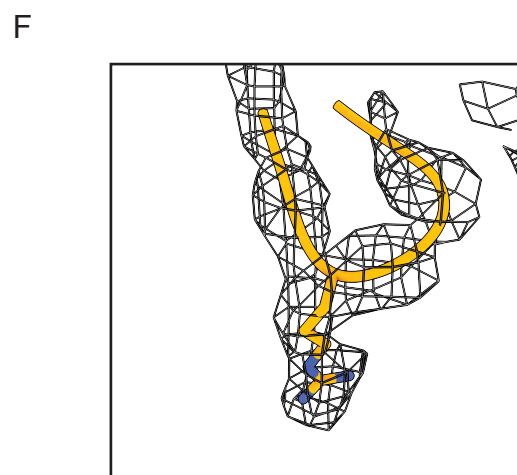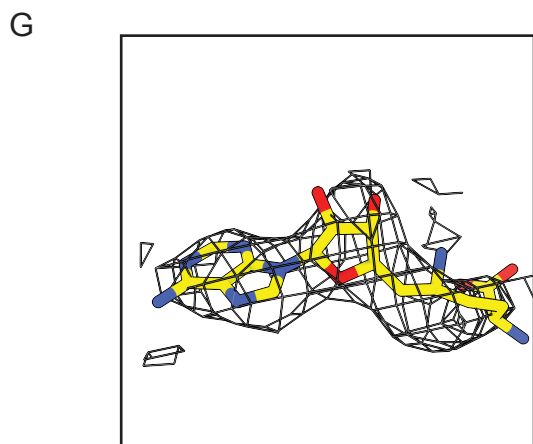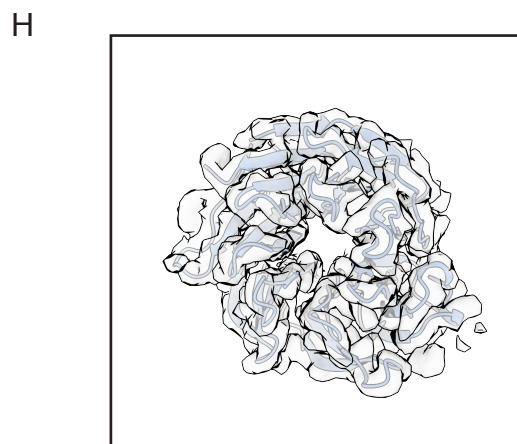

Supplement: Supplemental Figure 2 [file mmc4.pdf]

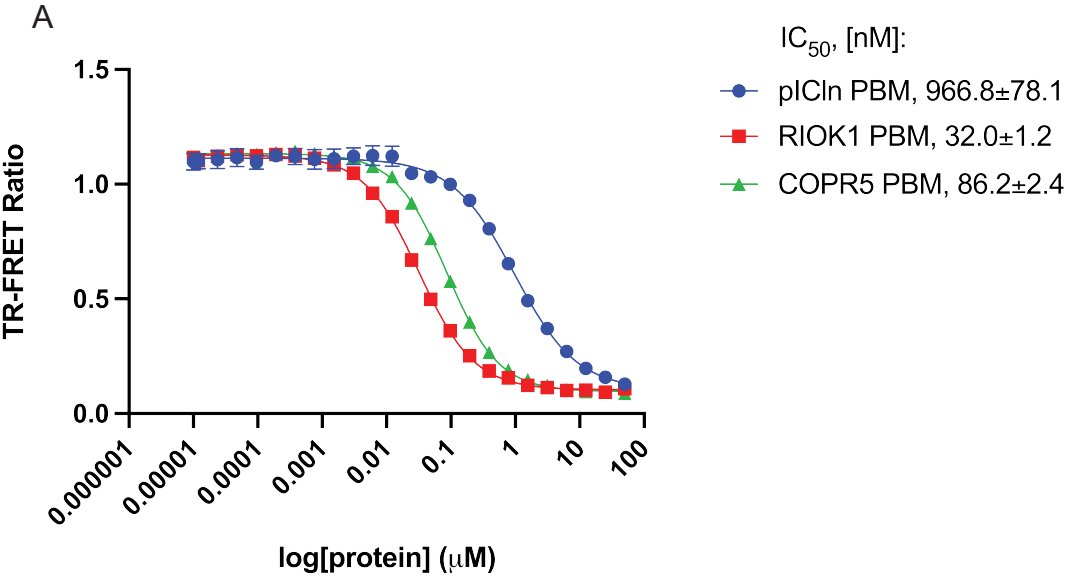

pICln PBM: TVAGQFEDADVDH  
RIOK1 PBM: VVPGQFDDADSSD  
COPR5 PBM: FETGQFDDAED

Supplement: Supplemental Figure 3 [file mmc5.pdf]

A

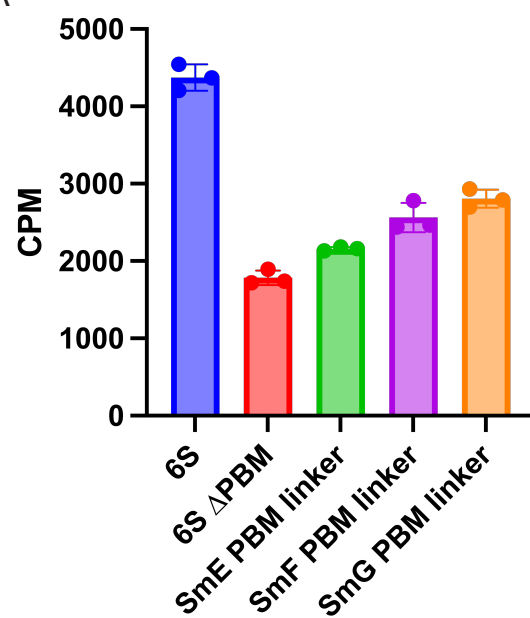

Supplement: Supplemental Figure 4 [file mmc6.pdf]
